# Registry Forge: an open-source end-to-end pipeline for patient-directed SMART on FHIR registries

**DOI:** 10.1093/jamiaopen/ooag200

**Published:** 2026-10-16

**Authors:** Danielle Boyce, Alan Premasiri, Shawn Sullivan, Beth Levine, Fernando G Vieira

**Affiliations:** ALS Therapy Development Institute, Watertown, MA 02472, United States; Biomedical Informatics and Data Science Section, Johns Hopkins University, Baltimore, MD 21205, United States; ALS Therapy Development Institute, Watertown, MA 02472, United States; ALS Therapy Development Institute, Watertown, MA 02472, United States; ALS Therapy Development Institute, Watertown, MA 02472, United States; ALS Therapy Development Institute, Watertown, MA 02472, United States

**Keywords:** patient registries, SMART on FHIR, Hl7, electronic health records, amyotrophic lateral sclerosis

## Abstract

**Objectives:**

Patient-directed SMART on FHIR lets registries acquire longitudinal electronic health record data, but the payload requires substantial engineering before use. We present Registry Forge, an open-source pipeline that converts it into curation-ready outputs.

**Materials and methods:**

Registry Forge parses C-CDA, HTML, RTF, PDF, and FHIR inputs, joins records to a canonical identifier, and emits a browser dashboard, an OMOP CDM v5.4 data set, GA4GH Phenopackets v2, a code inventory, and disease-specific narrative extractions.

**Results:**

Applied to the ALS Research Collaborative Study (94 participants, 56 US health systems), it processed 22 686 files and 1791 FHIR Bundles (109 599 resources); only 15.0% of files were full C-CDA XML; HTML (48.4%) and RTF (34.3%) predominated.

**Discussion:**

Payloads are far more heterogeneous than often assumed; the architecture is designed for extension, though generalizability remains to be demonstrated.

**Conclusion:**

Registry Forge closes the gap between acquisition and curation-ready data with no server infrastructure and is openly available.

## Introduction

The Substitutable Medical Applications, Reusable Technologies (SMART) on FHIR specification describes how a third-party application authenticates to a patient’s electronic health record (EHR) account and retrieves clinical data on the patient’s behalf using OAuth 2.0.^1^^,^^2^ Following the 21st Century Cures Act Final Rule, certified health IT modules that meet the ONC standardized API criterion must support FHIR R4 APIs and SMART App Launch and OpenID Connect authorization for patient-facing access.^3^^,^^4^ A patient can therefore authorize an external registry to retrieve available clinical data exposed through a compliant patient-facing API, reducing the need for separate site-by-site data use agreements.

Acquiring data is not the same as having usable data. In our patient-directed retrieval workflow, FHIR Bundle exports and DocumentReference and Binary retrieval yielded a mixture of Consolidated Clinical Document Architecture (C-CDA) XML, FHIR JSON, and narrative attachments in vendor-dependent formats.^5^^,^^6^^,^^7^ Each artifact must be decoded, parsed, deduplicated, joined to a canonical patient identifier, and rendered for review. Small registries need outputs that registry leaders, clinical coordinators, curators, and investigators can read directly without running code, which argues for a deliberately wide, extract-all-curate-later pipeline coupled to a viewer the whole team can use.

## Objectives

To produce a reproducible, local-first, open-source pipeline that (1) decodes and parses every artifact in a SMART on FHIR-acquired clinical document repository, (2) joins records across C-CDA and FHIR via a canonical patient identifier, (3) enriches incomplete display-name fields, (4) supports auditable exclusion of test records, (5) produces an OMOP Common Data Model (CDM) v5.4 data set tagged by Athena vocabulary release, (6) produces GA4GH Phenopackets v2 documents, and (7) recovers disease-specific content from unstructured note narratives. The intended context of use is observational patient registries and natural history studies, particularly rare disease registries operating with limited data engineering capacity.

## Methods

### Inputs and architecture

Two parallel directories on local disk were processed: 22 686 document files retrieved via SMART on FHIR DocumentReference and Binary endpoints, and 1791 JSON files each containing a FHIR Bundle for 1 patient. Registry Forge is organized as a 7-stage core that emits a single JSON bundle, plus independent add-on modules that consume that bundle (Figure 1). The separation means an adopting registry that only needs a viewer runs only the core; adopters mix and match add-ons by use case. The pipeline follows an extract-all, curate-later philosophy: the same observation may appear in both C-CDA and FHIR streams, and rather than deduplicate aggressively at ingest, the bundle preserves both rows with source-system provenance tags so adopters apply their own rules downstream.

**Figure 1.**
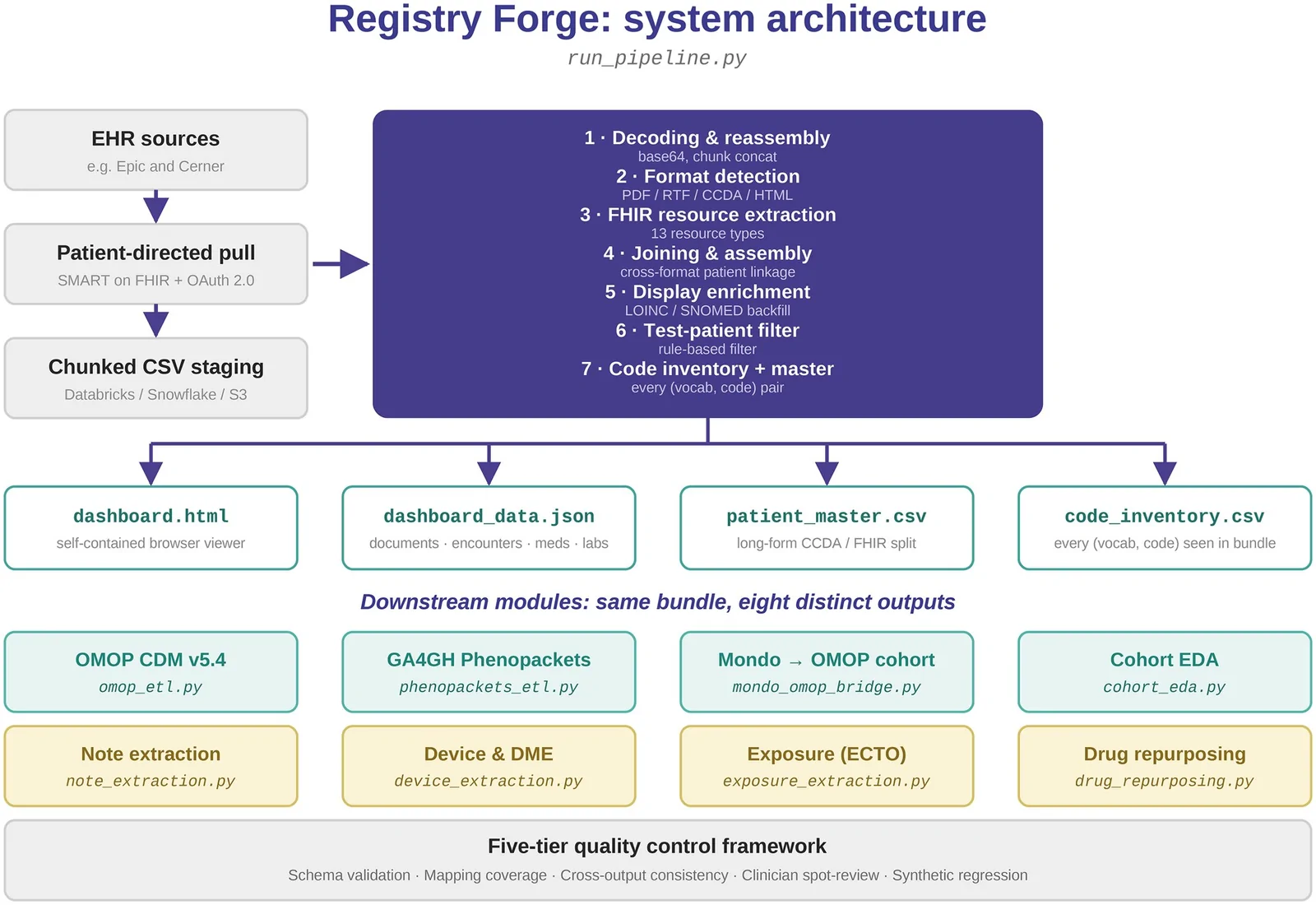
End-to-end pipeline architecture. The pipeline is split into a 7-stage core (Stages 1-7) that produces the bundle, code inventory, and browser viewer, plus a set of downstream add-on modules (OMOP CDM v5.4 ETL, GA4GH Phenopackets v2 ETL, Mondo-to-OMOP cohort bridge, regex-based note extraction, device/equipment extraction, exposure extraction, drug-repurposing signal extraction, and PHI-free cohort EDA report), each of which consumes the same bundle. All layers run on commodity hardware with no server infrastructure.

### Core pipeline

Stage 1 reassembles chunked CSV exports by document identifier and base64-decodes payloads with up to 2 passes. Stage 2 routes each file to a format-specific parser based on magic-byte detection: xml.etree.ElementTree for C-CDA, a custom stripper for RTF, pypdf for PDFs, and html.unescape with tag removal for HTML. A per-domain code prioritization (RxNorm > SNOMED > NDC for medications; SNOMED > ICD-10-CM for problems; LOINC >SNOMED for labs) selects the primary code while the complete coding list is preserved. Stage 3 walks each FHIR Bundle and dispatches each resource to a type-specific extractor across 13 resource types, resolving patient linkage via subject.reference. Stage 4 unifies structured tables by patient identifier, tags each row source = ccda or source = fhir, and emits the JSON bundle consumed by a static HTML dashboard (Figure 2). Stage 5 backfills missing displayName attributes from a built-in lookup of common LOINC codes. Stage 6 excludes test patient records via a configuration-driven filter supporting 4 match types. Stage 7 emits a code inventory listing each unique (vocabulary, code) pair with reference counts and patient counts. Files whose format cannot be identified are decoded as plain text, flagged as unknown, tallied in the run log, and retained for review rather than silently dropped; resource types outside the 13 supported extractors are skipped without aborting the run.

**Figure 2.**
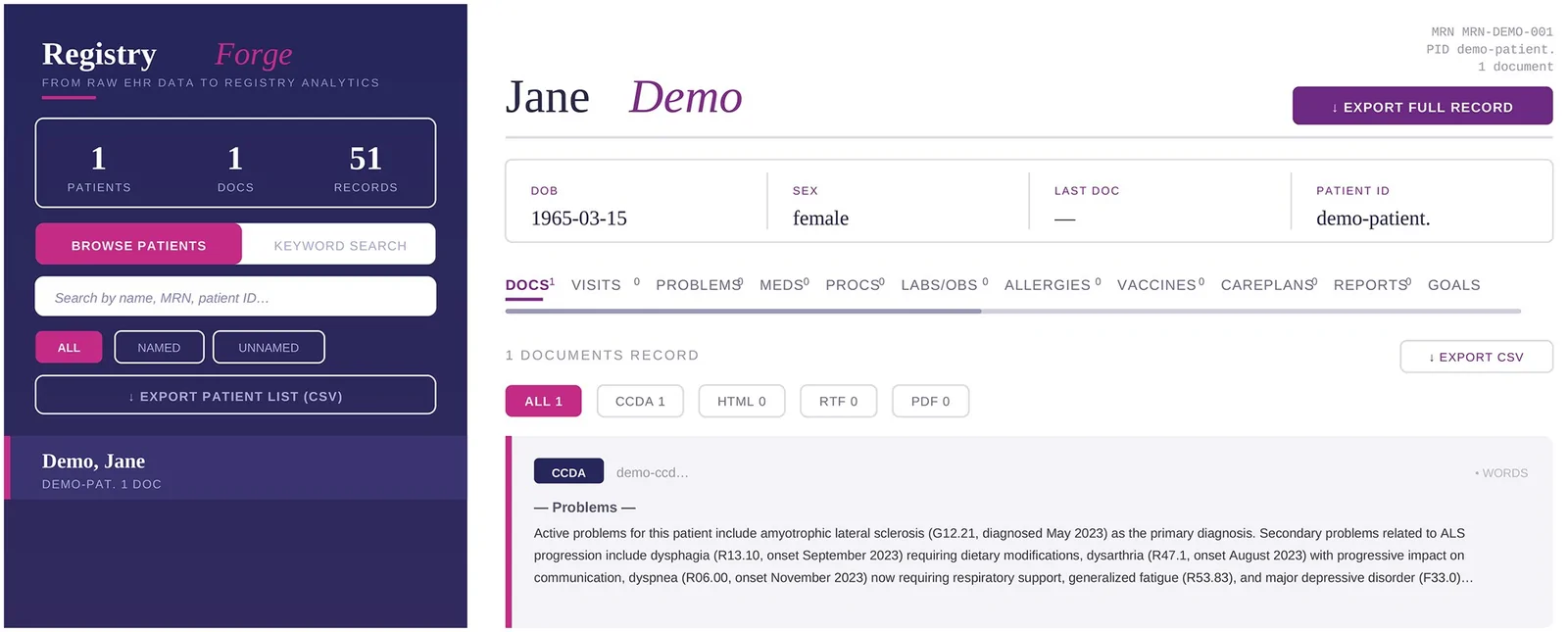
Browser-viewable participant dashboard. The single-file HTML dashboard renders the merged C-CDA plus FHIR bundle entirely client-side. Per-tab CSV-export buttons preserve the source provenance tags (source = CCDA or source = FHIR) for downstream analytic workflows.

### Add-on modules

An OMOP ETL reads the bundle plus an OHDSI Athena vocabulary download and writes 9 CDM v5.4 tables (PERSON, OBSERVATION_PERIOD, VISIT_OCCURRENCE, CONDITION_OCCURRENCE, DRUG_EXPOSURE, PROCEDURE_OCCURRENCE, MEASUREMENT, OBSERVATION, and CDM_SOURCE), routing records by the domain_id of the standard concept rather than by source category.^8^^,^^9^ A Phenopackets v2 ETL produces 1 JSON document per patient plus a Cohort document, with seed mappings for motor neuron disease, epilepsy, and autoimmune neurologic disease.^10^ A regex note-extraction module applies a pattern library to every free-text source and emits 1 row per match with patient identifier, source, pattern, value, a snippet, and offset. Shipped ALS-specific patterns target ALSFRS-R scores, El Escorial certainty, site of onset, family history, gene mentions, forced vital capacity, and treatment milestones. The patterns are seed patterns for site-specific tuning, not validated production NLP; sites with mature NLP should use cTAKES,^11^ medspaCy,^12^ or transformer-based models. Additional modules are documented in the public repository.

## Results

We applied the pipeline to the ALS Research Collaborative Study EHR cohort (*n* = 94 participants).^13^ We attributed records to source systems using the FHIR service endpoint and resource metadata (Bundle.meta.source and entry.fullUrl), resolving named institutions where possible. Records resolved to 56 US health systems, predominantly Epic with some Cerner (Table 1). Generic Epic-hosted and Cerner tenant endpoints that could not be mapped to a specific institution were retained as unresolved source-system links. About one third of participants linked records from more than 1 system. Participants arriving without a name or source system in structured fields are flagged for review in the viewer; in our experience these details are often recoverable from the unstructured notes and can be corrected after import.

**Table 1.** Health systems represented in the ALS research collaborative study cohort, attributed from the FHIR service endpoint.

Health system	EHR vendor	Participants
**Mass General Brigham**	Epic	9
**Duke University Health**	Epic	8
**Mayo Clinic**	Epic	6
**Houston Methodist**	Epic	4
**Keck Medicine of USC**	Cerner	4
**Henry Ford Health**	Epic	3
**UCSF**	Epic	3
**Kaiser Permanente**	Epic	3
**Johns Hopkins Health System**	Epic	2
**NYU Langone Health**	Epic	2
**Penn Medicine**	Epic	2
**Northwestern Medicine**	Epic	2
**University of Michigan**	Epic	2
**UCHealth**	Epic	2
**Dartmouth Health**	Epic	2
**University of Rochester**	Epic	2
**Atrius Health**	Epic	2
**St. Elizabeth Healthcare**	Epic	2
**Additional named systems, 1 participant each (*n* = 38)^a^**	Epic/Cerner	38

Counts are distinct participants per system and are non-exclusive; some participants linked records from more than 1 system, so the column sums to more than the 94 distinct participants.

a The 38 single-participant systems were: Allegheny Health Network, Allina Health, Atrium Health Wake Forest Baptist, Beth Israel Lahey Health, Bozeman Health, Cambridge Health Alliance, Cedars-Sinai, Centura Health, Charleston Area Medical Center, Cleveland Clinic, Corewell Health, FirstHealth, Froedtert Health, Guthrie, Jefferson Health, M Health Fairview, Mount Sinai Health System, NorthShore (Endeavor Health), Northwell Health, Ochsner Health, Ohio State University Wexner Medical Center, Overlake Medical Center, Parkview Health, Providence, SCL Health, SSM Health, Saint Francis Health System, Sturdy Memorial Hospital, Sutter Health, Temple Health, Trinity Health, University of Kansas Health System, UC Davis Health, UCLA Health, UNC Health, UVA Health, UW Medicine, Vanderbilt.

Magic-byte format detection at Stage 2 revealed that only 15.0% of the 22 686 assembled files were full C-CDA XML documents; HTML narrative fragments (48.4%) and RTF clinical notes (34.3%) together comprised more than four-fifths of the repository, with PDF documents (2.3%) making up the remainder. A registry that assumes all DocumentReference attachments are C-CDA would silently drop the majority of its source material.

Across all sources, the pipeline processed 109 599 FHIR resources from the Bundle stream and extracted 33 084 C-CDA section narratives plus 14 825 narrative documents after test-record exclusion (791 records associated with 2 confirmed test patients were removed). The Stage 7 code inventory enumerated several thousand unique (vocabulary, code) pairs spanning SNOMED CT, ICD-10-CM, RxNorm, LOINC, CVX, and CPT-4. Approximately 36% of LOINC-coded observations in source C-CDA documents carried no displayName attribute. Because CDA displayName values are human-readable aids rather than the semantic source of truth, Registry Forge backfills labels for review while retaining the original code and provenance; this complements broader work on LOINC code and unit harmonization in real-world data.^14^ Stage 5 enrichment populated 39 022 labels, raising display-name coverage from 64% to 84%. The OMOP ETL successfully populated all 9 target CDM tables, and CDM_SOURCE.csv recorded the exact vocabulary versions used. The Phenopackets ETL produced 1 valid v2 JSON document per participant. On the synthetic demonstration cohort, the regex-based note-extraction module recovered every category of ALS-specific content patterned in the shipped library (Figure 3).

**Figure 3.**
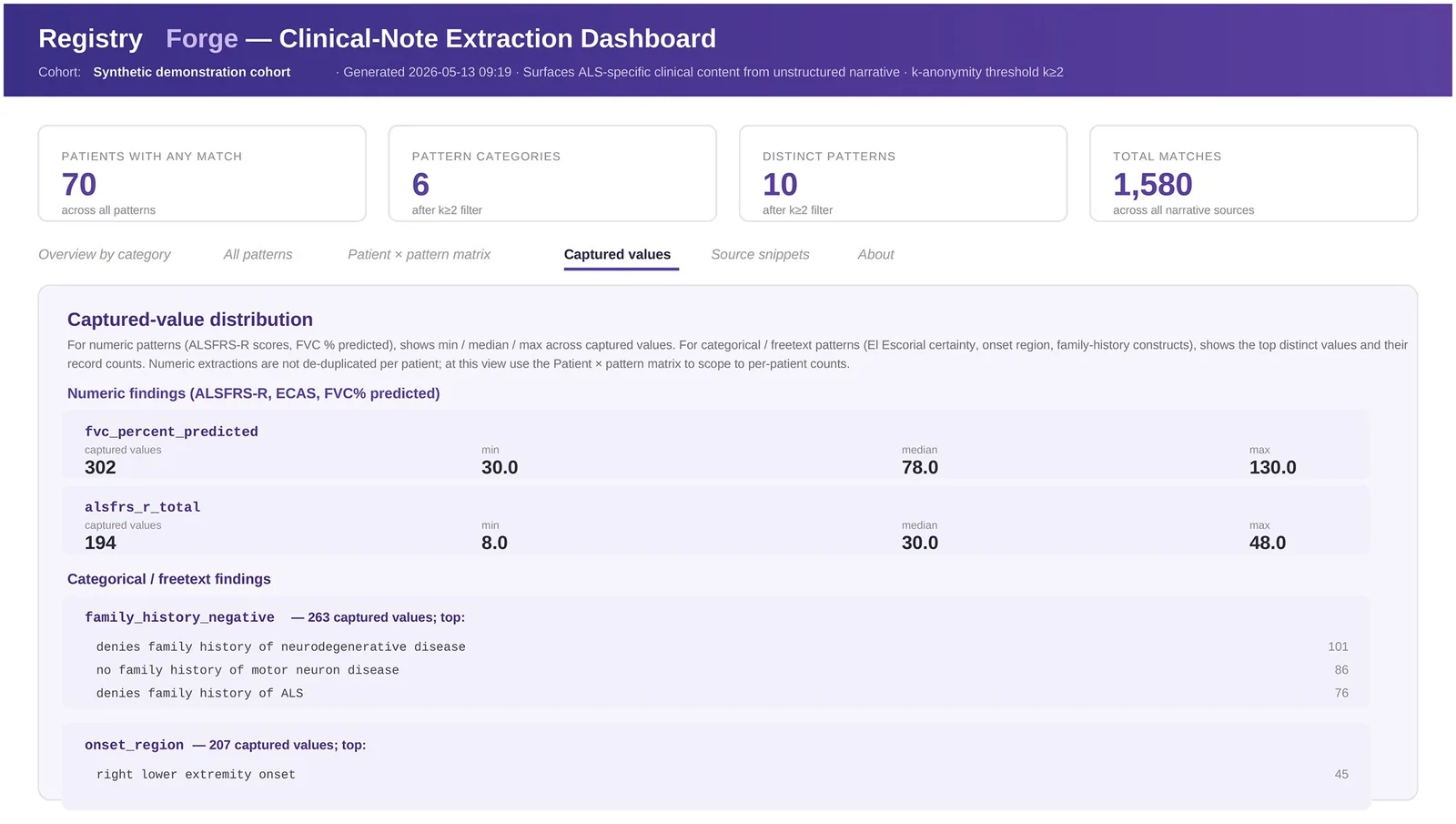
Regex-based recovery of unstructured clinical content run against a synthetic cohort. Output rows from the note-extraction module run against a synthetic Plan-of-Care narrative. Every row carries a ± 60-character snippet for manual review; pattern name and description allow rapid grouping of matches by clinical concept.

Operationally, a complete end-to-end run over the full repository takes approximately 30 minutes on a standard laptop (Windows 11, 32 GB RAM) with no server or cloud infrastructure, and the OMOP ETL adds approximately 5 minutes; routine manual intervention is limited to reviewing participants flagged as unnamed in the viewer and maintaining the test-patient exclusion list.

A fully synthetic single-patient interactive demonstration of the dashboard, exercising every record category with codes verified against RxNav, SNOMED CT, ICD-10-CM, LOINC, CVX, and CPT-4, is publicly hosted alongside the source code at https://alstdi.github.io/RegistryForgeALS/ to allow other registries to evaluate the pipeline before adopting it.

## Discussion

The challenges of extracting and processing patient-directed EHR data are not unique to ALS or the ALS Research Collaborative Study. Registries acquiring data through patient-directed SMART on FHIR are likely to face structurally similar problems, and the broader literature on secondary use of EHR data has documented them across disease areas.^15^^,^^16^  Table 2 summarizes the common patterns we encountered and the corresponding pipeline layer that addresses each. What needs to be customized per registry is well-defined: a thin acquisition adapter to populate the 2 input directories; patient-identifier mapping queries against the registry’s own warehouse; disease-specific display-name lookups and regex patterns; and seed phenotype/disease tables for adopters using the Phenopackets module.

**Table 2.** Common post-acquisition problems observed in this study, likely to face other patient-directed SMART on FHIR registries, and the Registry Forge layer that addresses each.

Common problem	Registry Forge layer	Adapter effort
**Heterogeneous payload formats (HTML/RTF/PDF behind DocumentReference)**	Stage 2 magic-byte detection plus format-specific parsers	None; works as shipped
**Identifier-mapping provenance gaps**	Stage 4 join against patient-import audit table	Adopter writes SQL against own warehouse
**Vendor-specific display-name omissions**	Stage 5 LOINC/SNOMED display-name backfill	Extend lookup table for disease area
**Test or non-clinical records**	Stage 6 configuration-driven filter (4 match types)	None; works as shipped
**Source codes unknown to mapping pipeline**	Stage 7 code inventory CSV	None; works as shipped
**OMOP vocabulary version drift across runs**	Vocabulary-tagged output folder plus CDM_SOURCE.csv versions	None; works as shipped
**Disease-specific content trapped in narrative**	Note-extraction module (regex library)	Author site-specific patterns

Adapter effort indicates the work an adopting registry can expect to do for that pipeline layer beyond plugging the pipeline into the registry’s own acquisition output.

The most broadly applicable finding of this study may be the payload composition itself: only 15.0% of retrieved artifacts were complete C-CDA documents, while HTML and RTF narrative artifacts comprised more than four-fifths of the repository. Real world patient-directed data are far more heterogeneous than many investigators expect, and a pipeline that assumes C-CDA input will discard most of its source material. Prior FHIR-based phenotyping work is typically developed against a single institution or a curated research corpus rather than the heterogeneous multi-vendor payload returned by patient-facing endpoints;^6^ prior common data model work assumes source data already in relational form.^9^ Registry Forge instead ingests the full payload actually returned by patient-directed SMART on FHIR retrieval.

A related distinction is between interoperability and harmonization. Registry Forge brings heterogeneous artifacts into a common, provenance-tagged framework; its outputs are curation-ready rather than analysis-ready. Reconciling duplicate observations arriving through both C-CDA and FHIR streams, conflicting values, temporal alignment across systems, phenotype definition, and cohort construction remain downstream activities requiring methodological judgment. Our results demonstrate engineering validation (successful parsing, linkage, and population of standard output formats) rather than semantic validation: populated OMOP tables and schema-valid Phenopackets do not by themselves establish concept-mapping accuracy or preservation of clinical meaning, which require study-specific evaluation such as clinician review.

Acquisition is likewise only 1 component of registry operations. Adopters must still manage patient identity and longitudinal linkage across refreshes, governance, incomplete institutional participation, and variability across vendor FHIR implementations in profiles, optional elements, extensions, and terminology practice. Registry Forge tolerates much of this variability by design: missing optional elements are treated as absent rather than fatal, codes from any vocabulary flow to the Stage 7 inventory, and unsupported resource types are skipped. It does not, however, normalize semantic differences between implementations.

These boundaries clarify who stands to benefit most. Health systems can query their EHR directly and may need only the downstream modules. The core audience is registries operated outside health systems, including patient advocacy nonprofits that acquire records through commercial patient-directed retrieval services: they often receive the raw payload characterized here, rarely have the engineering staff to transform it, and can run Registry Forge end-to-end with basic Python skills.

Registry Forge has been developed and tested against data from a single registry and validated end-to-end on a fully synthetic demonstration cohort; validation against data sets from other registries and EHR vendors is invited. The present evaluation demonstrates feasibility within 1 implementation context rather than generalizability across diseases, registry types, or FHIR environments; statements about broader applicability describe anticipated extensibility rather than demonstrated performance. The production cohort reported here is small and reflects the early adoption phase of the ALS Research Collaborative Study’s SMART on FHIR onboarding; the patterns described should be read as engineering generalizations rather than as epidemiologic claims about ALS. The shipped regex pattern library is a seed for site-specific tuning, not validated production NLP. The 5-tier quality-control framework included with the pipeline is a starting set and is not a substitute for the Kahn et al. data-quality framework^17^ or the OHDSI Data Quality Dashboard.^18^ Registry Forge automates the engineering work of registry data integration but does not replace the clinical, methodological, and editorial judgment adopting organizations must apply to their own data.

## Conclusion

Patient-directed SMART on FHIR has substantially expanded the data acquisition options available to patient registries, but the analytics work required to make acquired data usable falls disproportionately on small registries lacking in-house data engineering capacity. In the cohort reported here, that payload was dominated by narrative artifacts rather than complete C-CDA documents. Within the context demonstrated here, Registry Forge closes this gap end-to-end with no server infrastructure, converting a heterogeneous SMART on FHIR-acquired document repository into a researcher-usable browser viewer, an OMOP CDM v5.4 data set, GA4GH Phenopackets, a code inventory, and structured extractions of disease-specific narrative content. The full implementation, including a fully synthetic demonstration data set, is publicly available at https://alstdi.github.io/RegistryForgeALS.

A further 26 participant-system links resolved to Epic generic-hosting tenants (hosts of the form etNNNN.epichosted.com) or Cerner tenants whose specific institution could not be determined from the endpoint URL or the organization names embedded in the data; these are not listed individually. Across the cohort, records resolved to 56 distinct named US health systems.

## Data Availability

Source code for the complete pipeline, the browser dashboard, and a fully synthetic single-patient demonstration cohort are publicly available at https://alstdi.github.io/RegistryForgeALS/ (repository: https://github.com/alstdi/RegistryForgeALS) under the MIT License. The exact release used for this manuscript is archived with a citable DOI (https://doi.org/10.71944/2P5C-NG50) through ALS TDI’s DataCite membership. The clinical data described in this manuscript cannot be openly shared due to the terms of the ALS Research Collaborative Study informed consent. A de-identified version of the ALS Research Collaborative Study data is available to qualified researchers through the ARC Data Commons (https://www.als.net/arc/data-commons/).

## References

[1] Mandel JC , Kreda DA, Mandl KD, et al SMART on FHIR: a standards-based, interoperable apps platform for electronic health records. J Am Med Inform Assoc. 2016;23:899-908. 10.1093/jamia/ocv189 PMC4997036 26911829

[2] Bender D , Sartipi K. HL7 FHIR: an agile and RESTful approach to healthcare information exchange. Proc IEEE Int Symp Comput Based Med Syst. 2013:326-331.

[3] Office of the national coordinator for health information technology. 21st century cures act: interoperability, information blocking, and the ONC health IT certification program. Fed Regist. 2020;85:25642-25961.

[4] HL7 International. SMART App Launch Implementation Guide. HL7 FHIR. Accessed May 2026. https://hl7.org/fhir/smart-app-launch/

[5] D’Amore JD , Mandel JC, Kreda DA, et al Are meaningful use stage 2 certified EHRs ready for interoperability? Findings from the SMART C-CDA collaborative. J Am Med Inform Assoc. 2014;21:1060-1068. 10.1136/amiajnl-2014-002883 PMC4215060 24970839

[6] Hong N , Wen A, Stone DJ, et al Developing a FHIR-based EHR phenotyping framework: a case study for identification of patients with obesity and multiple comorbidities. J Biomed Inform. 2019;99:103310. 10.1016/j.jbi.2019.103310 PMC6990976 31622801

[7] HL7 International. US Core Implementation Guide: Document Reference Profile. HL7 FHIR. Accessed May 2026. https://hl7.org/fhir/us/core/StructureDefinition-us-core-documentreference.html

[8] Observational Health Data Sciences and Informatics. OMOP Common Data Model v5.4 specification. Accessed May 2026. https://ohdsi.github.io/CommonDataModel/cdm54.html

[9] Voss EA , Makadia R, Matcho A, et al Feasibility and utility of applications of the common data model to multiple, disparate observational health databases. J Am Med Inform Assoc. 2015;22:553-564. 10.1093/jamia/ocu023 PMC4457111 25670757

[10] Jacobsen JOB , Baudis M, Baynam GS, GAGH Phenopacket Modeling Consortium, et al The GA4GH phenopacket schema defines a computable representation of clinical data. Nat Biotechnol. 2022;40:817-820. 10.1038/s41587-022-01357-4 PMC9363006 35705716

[11] Savova GK , Masanz JJ, Ogren PV, et al Mayo clinical text analysis and knowledge extraction system (cTAKES): architecture, component evaluation and applications. J Am Med Inform Assoc. 2010;17:507-513. 10.1136/jamia.2009.001560 PMC2995668 20819853

[12] Eyre H , Chapman AB, Peterson KS, et al Launching into clinical space with medspaCy: a new clinical text processing toolkit in python. AMIA Annu Symp Proc. 2021;2021:438-447. PMC8861690 35308962

[13] Boyce D , Premasiri A, Huynh NPT, et al The ALS research collaborative: a long-running multimodal ALS natural history resource. Res Sq. 2025. 10.21203/rs.3.rs-8272744/v1 Preprint.

[14] Naliyatthaliyazchayil P , Stenerson T. Harmonizing logical observation identifiers names and codes (LOINC) codes and units in real-world oncology data: method development and evaluation. JMIR Med Inform. 2026;14:e81254. 10.2196/81254 PMC13010070 41802234

[15] Hripcsak G , Duke JD, Shah NH, et al Observational health data sciences and informatics (OHDSI): opportunities for observational researchers. Stud Health Technol Inform. 2015;216:574-578. PMC4815923 26262116

[16] Kohane IS , Aronow BJ, Avillach P, et al; Consortium For Clinical Characterization of COVID-19 by EHR (4CE). What every reader should know about studies using electronic health record data but may be afraid to ask. J Med Internet Res. 2021;23:e22219. 10.2196/22219 PMC7927948 33600347

[17] Kahn MG , Callahan TJ, Barnard J, et al A harmonized data quality assessment terminology and framework for the secondary use of electronic health record data. EGEMS (Wash DC). 2016;4:1244. 10.13063/2327-9214.1244 PMC5051581 27713905

[18] Blacketer C , Defalco FJ, Ryan PB, Rijnbeek PR. Increasing trust in real-world evidence through evaluation of observational data quality. J Am Med Inform Assoc. 2021;28:2251-2257. 10.1093/jamia/ocab132 PMC8449628 34313749

